# The complete mitochondrial genome sequence of *Triplophysa stenura* (Teleostei, Cypriniformes): genome characterization and phylogenetic analysis

**DOI:** 10.1080/23802359.2016.1209093

**Published:** 2016-09-05

**Authors:** Yongliu Yan, Dehuai Luo

**Affiliations:** School of Life Sciences, Southwest University, Chongqing, China

**Keywords:** Mitochondrial genome, phylogenetic relationship, *Triplophysa stenura*

## Abstract

The nemacheilid genus *Triplophysa* is widely distributed in the Qinghai-Tibet Plateau and its adjacent areas. The complete mitochondrial genome of the loach *Triplophysa stenura* collected from the Peigucuo Lake was sequenced. The genome was 16,569 bp in length, including 13 protein-coding genes, 22 transfer RNA genes, two ribosomal RNA genes, and a non-coding control region (D-loop). Most of the genes were encoded on the heavy strand, except for *ND6* and eight tRNA genes. The overall base composition of *T. stenura* was 27.8% for A, 28.4% for T, 25.4% for C, 18.4% for G, with a slight A + T rich feature (56.2%). Phylogenetic analyses showed that all *Triplophysa* species clustered together and *T. stenura* formed a sister relationship with *T. tibetana*. The complete mitogenome data of *T. stenura* would provide essential information in understanding phylogenetic relationships among *Triplophysa* species.

The genus *Triplophysa* is a strongly diverged fish group in the family Nemacheilidae, with 140 valid species, more than 80% of which are known from China so far (Ren et al. 2012; Froese & Pauly 2016). However, less than 20 complete mitogenome sequences of this genus are available from GenBank until now. *Triplophysa stenura* occurs in the Qinghai-Tibet Plateau, upper Yangtze, Mekong, Salween and Brahmaputra drainages (Kottelat 2012; Zhang et al. 2015). Here we determined the complete mitochondrial genome sequence of *T. stenura* (16569bp; GenBank accession number KX354975) and analyzed its phylogenetic position within genus *Triplophysa*.

Samples of *T. stenura* were collected from Peigucuo Lake (29°1′N, 85°34′13″E), a tributary of the Yarlung Zangbo River in Tibet, and preserved in the Key Laboratory of Freshwater Fish Reproduction and Development (Southwest University, China). We designed eight sets of primers and methods for sequence analyses strategies followed the procedures outlined in Wang et al. (2012).

The complete mitogenome of *T. stenura* was a circular molecule with 16,569 nucleotides, including 13 protein-coding genes, 2 ribosomal RNA genes, 22 tRNA genes, and a non-coding control region (D-loop), which demonstrated a typical vertebrate mitogenome feature (Peng et al. 2006). Most of the genes were encoded on the heavy strand, except for *ND6* and eight tRNA genes (tRNA^Gln^, tRNA^Ala^, tRNA^Asn^, tRNA^Cys^, tRNA^Tyr^, tRNA^Ser(UCN)^, tRNA^Glu^ and tRNA^Pro^). The rank of overall nucleotide composition was T (28.4%)>A (27.8%)>C (25.4%)> G (18.4%), and A + T content (56.17%) was higher than G + C content (43.83%). All of the 13 protein-coding genes showed the regular initiation codon ATG with the sole exception of *COI* (started with GTG). Furthermore, the stop codons for five of the 13 protein-coding genes were TAA (*ND1*, *COI*, *ATP6*, *ATP8*, *ND4L*) and two of them were TAG (*ND5, ND6*), whereas other six genes possessed incomplete stop codons TA or T (*ND2*, *ND3*, *ND4*, *COII*, *COIII* and *cytb*). The gene arrangements were similar with some other *Triplophysa* species (e.g. Lei et al. 2016).

In order to explore the phylogenetic position of *T. stenura*, we retrieved other available mitogenome sequences of *Triplophysa* species and constructed a neighbour-joining tree in MEGA 6.0 (Tamura et al. 2013). The result strongly supported that *T. stenura* formed a sister relationship with *T. tibetana* (Figure 1). Therefore, we expect that our new mitogenome data of *T. stenura* will contribute to elucidate the phylogenetic relationships among all the species of *Triplophysa*.

**Figure 1.**
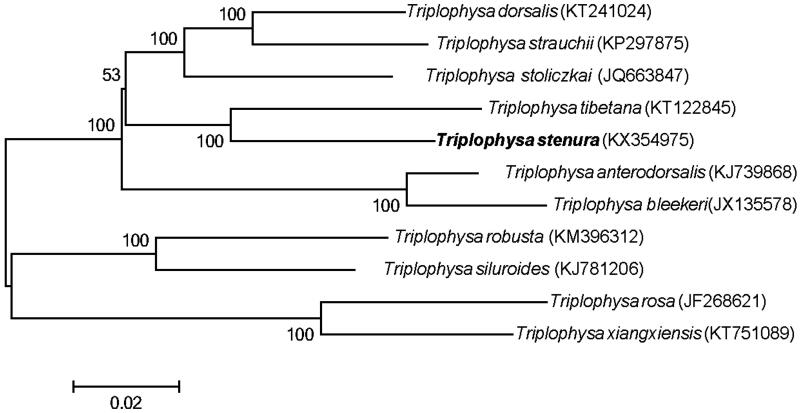
Neighbour-joining phylogenetic tree of *Triplophysa stenura* and other 10 species of *Triplophysa* based on complete mtDNA sequences.
